# Urban Animal Exposures and Rabies Post-Exposure Prophylaxis in Istanbul, Turkey: Insights from a Metropolitan Emergency Department

**DOI:** 10.3390/tropicalmed11040107

**Published:** 2026-04-20

**Authors:** Cansel Askin, Behcet Al, Cihad Unsal Karahaliloglu, Yunus Emre Gemici, Ibrahim Coban, Abdulkerim Emre Yanar

**Affiliations:** Emergency Department, Faculty of Medicine, İstanbul Medeniyet University, Göztepe Prof. Dr. Süleyman Yalçın City Hospital, 34722 İstanbul, Türkiye; canselaskin95@gmail.com (C.A.);

**Keywords:** rabies, urban health, animal exposure, cat-related injuries, post-exposure prophylaxis, emergency department

## Abstract

Background: Rabies remains a major zoonotic disease worldwide, particularly in regions with large populations of free-roaming animals. In urban settings, animal-related injuries constitute a substantial healthcare burden and frequently result in the administration of rabies post-exposure prophylaxis (PEP). This study aimed to evaluate the epidemiological characteristics of animal exposures and real-world PEP practices in a metropolitan emergency department. Methods: This retrospective descriptive study included 1960 patients presenting to a tertiary metropolitan emergency department between 1 March and 1 September 2025 with suspected animal exposure. Demographic data, animal species involved, exposure mechanisms, animal ownership and vaccination status, time to presentation, and PEP practices were analyzed using descriptive statistics. Results: Most exposures were cat-related (86.3%) and were caused by scratching (81.5%). Nearly all injuries were superficial (99.8%), while deep injuries were rare (0.2%). The majority of animals were classified as strays (90.1%), and vaccination status was unknown in 81.2% of cases. Rabies immunoglobulin was administered to only 0.6% of patients; however, rabies vaccination was initiated in 98.8% of patients. Approximately 74.5% of patients presented within 24 h. Post-exposure animal observation was documented in only 20.2% of cases. Conclusions: Urban animal exposures in this metropolitan setting were predominantly superficial and cat-related, yet nearly all patients received rabies vaccination. Limited animal observation and incomplete vaccination documentation appear to constrain risk stratification and may contribute to the use of precautionary PEP. Strengthening surveillance systems, improving documentation, and implementing evidence-based risk-stratification strategies are essential for optimizing rabies prophylaxis practices in urban environments.

## 1. Introduction

Rabies is a zoonotic viral infection transmitted through contact of infected animal saliva with broken skin or mucous membranes and, if left untreated, is almost invariably fatal [1,2,3]. Caused by lyssaviruses, rabies leads to rapidly progressive encephalomyelitis of the central nervous system [3]. Once clinical symptoms develop, the mortality rate approaches 100%, making rabies one of the most lethal infectious diseases worldwide [4]. Despite being entirely preventable through timely and appropriate post-exposure prophylaxis (PEP), rabies continues to pose a significant global public health challenge. The majority of human rabies deaths occur in Asia and Africa, where access to timely post-exposure prophylaxis is often limited. Children under 15 years of age account for a substantial proportion of rabies victims due to their frequent contact with animals.

According to the World Health Organization (WHO), approximately 59,000 people die from rabies annually, and more than 15 million individuals receive PEP each year following suspected exposure [5,6]. The global strategy to eliminate dog-mediated human rabies by 2030 highlights the importance of integrated surveillance, mass animal vaccination, and rational PEP administration [7,8]. Although the majority of rabies-related deaths occur in Asia and Africa, the burden of suspected exposure and subsequent prophylaxis extends well beyond confirmed cases, particularly in regions with dense populations of free-roaming animals and frequent human–animal interaction [6,9]. In such settings, the number of individuals seeking medical care for potential exposure may greatly exceed the number of confirmed infections, resulting in substantial healthcare utilization.

Rabies transmission most commonly occurs through bites; however, scratches and contamination of open wounds or mucosal surfaces may also pose a risk of infection [10,11]. Because rabies can only be prevented through timely PEP administration, accurate exposure assessment and strict adherence to evidence-based guidelines are essential [11,12]. In clinical practice, uncertainty about the animal’s vaccination status, limited post-exposure observation, and precautionary management approaches often influence decisions regarding prophylaxis. Consequently, patterns of PEP administration may not always directly correspond to the actual biological risk of transmission.

Türkiye remains at risk of rabies due to its geographic location, ecological diversity, and substantial population of free-roaming animals [13,14]. Each year, approximately 250,000–300,000 individuals present to healthcare facilities with suspected rabies exposure, most commonly following cat and dog injuries [13,15]. In large metropolitan areas, rapid urbanization and close human–animal coexistence have contributed to an increase in animal-related injuries [14,16]. In contrast to many endemic regions where dogs are the principal source of transmission, urban centers in Türkiye frequently report cat-related exposures as the dominant pattern [15,17]. The unique urban ecology of cities such as Istanbul—characterized by widespread free-roaming cats and daily human interaction in residential and public spaces—may significantly influence exposure dynamics.

Emergency departments serve as the primary point of care for individuals with suspected rabies exposure and play a central role in risk assessment and PEP initiation [13,18].

In this study, low-severity exposures generally referred to superficial scratches or minor injuries without deep-tissue involvement, broadly corresponding to WHO Category II exposures.

While precautionary prophylaxis is justified given the fatal nature of rabies, extensive PEP utilization in predominantly superficial injuries raises important questions regarding risk stratification, surveillance capacity, and optimal resource allocation [19,20,21].

Although several regional studies have evaluated animal bite characteristics in Türkiye, comprehensive data focusing specifically on urban exposure patterns and real-world PEP practices in large metropolitan emergency departments remain limited. Understanding how exposure severity, animal characteristics, and surveillance constraints shape prophylaxis decisions is essential for developing context-sensitive and evidence-based rabies prevention strategies.

In this context, we retrospectively evaluated patients presenting to the Emergency Department of Prof. Dr. Süleyman Yalçın City Hospital with animal-related injuries between 1 March and 1 September 2025. Demographic characteristics, the animal species involved, exposure mechanisms, animal vaccination and ownership status, time to presentation, and PEP practices were analyzed. This study aims to provide region-specific epidemiological data and to contribute to improved risk stratification and optimization of rabies post-exposure prophylaxis practices in metropolitan settings.

## 2. Methods

### 2.1. Study Design and Ethical Approval

This retrospective descriptive study was conducted at İstanbul Göztepe Prof. Dr. Süleyman Yalçın City Hospital, a tertiary metropolitan referral center. The study included all patients presenting to the Emergency Department between 1 March 2025, and 1 September 2025, with suspected animal exposure, defined as bites, scratches, or mucosal contact that required medical evaluation for potential rabies risk.

Ethical approval was obtained from the Clinical Research Ethics Committee of Prof. Dr. Süleyman Yalçın City Hospital (Decision No: 2025/0151; 11 September 2025). The study was observational, and no additional interventions were implemented. All data were anonymized prior to analysis. The study complied with the Declaration of Helsinki and national regulations governing clinical research in Türkiye [22,23].

### 2.2. Study Setting and Population

The study was conducted in collaboration with the Emergency Medicine Department and the Rabies Vaccination Unit of the Department of Infectious Diseases and Clinical Microbiology. In 2024, the Emergency Department recorded 375,950 patient visits, consistent with that of a high-volume urban referral center.

All consecutive patients presenting with suspected animal exposure during the study period were screened. Initial clinical evaluation was performed in the emergency department, and patients requiring rabies prophylaxis were subsequently referred to the Rabies Vaccination Unit.

During the study period, 2202 patient records were identified. After 242 cases (11%) were excluded due to incomplete records, duplicate entries for the same exposure event, or unverifiable exposure documentation, 1960 patients were included in the final analysis.

### 2.3. Data Collection

Data were extracted from the hospital’s electronic medical record system (Nucleus) and cross-checked with records from the Rabies Vaccination Unit. A standardized data collection form was used to ensure uniform data retrieval. No personally identifiable information was recorded. Records that did not meet the inclusion criteria or contained insufficient information were excluded from analysis. The number of recommended rabies vaccine doses was determined in accordance with national rabies prophylaxis guidelines of the Republic of Türkiye and World Health Organization recommendations, taking into account the characteristics of the exposure and the patient’s vaccination history.

### 2.4. Variables

The following variables were analyzed:

- Demographic characteristics (age, sex, occupation).
- Mode and location of presentation.
- Animal species involved.
- Mechanism and type of exposure.
- Animal ownership status (stray vs. owned).
- Animal vaccination status and post-exposure observation.
- Patient pre-exposure vaccination status.
- Administration of rabies vaccine and rabies immunoglobulin.
- Clinical outcome.

Animal exposure was defined as any bite, scratch, or contact with a mucous membrane that required medical assessment for the potential risk of rabies transmission. For descriptive purposes, children were defined as individuals younger than 18 years of age. The category “student” referred to individuals whose occupation was recorded as student in the hospital registration system, regardless of educational level. In this study, superficial injuries were generally defined as minor scratches or abrasions without deep tissue involvement, broadly corresponding to exposures similar to WHO Category II. Deep injuries were defined as transdermal bites or wounds involving deeper tissue layers, broadly corresponding to exposures similar to WHO Category III. Occupational categories were based on the patient’s occupation information recorded in the hospital registration system. The category “worker” referred to manual laborers, while “officer” referred to government employees (public sector workers).

### 2.5. Inclusion and Exclusion Criteria

All patients of any age presenting during the study period with suspected animal exposure were eligible for inclusion.

Exclusion criteria were:

- Incomplete or missing medical records.
- Duplicate visits related to the same exposure event.
- Unverifiable animal exposure.
- Presentations unrelated to suspected rabies risk.

### 2.6. Statistical Analysis

Statistical analyses were performed using IBM SPSS Statistics for Windows, Version 26.0 (IBM Corp., Armonk, NY, USA) [24].

Continuous variables were presented as mean ± standard deviation (SD) or median (minimum–maximum) as appropriate. Categorical variables were expressed as frequencies and percentages.

This study was reported in accordance with the Strengthening the Reporting of Observational Studies in Epidemiology (STROBE) guidelines.

## 3. Results

A total of 1960 patients presenting with suspected animal exposure between 1 March and 1 September 2025 were included in the final analysis. Results are presented under three main domains: demographic characteristics, exposure-related features, and post-exposure management.

### 3.1. Demographic Characteristics

Patient ages ranged from 10 months to 89 years, with a mean age of 28.79 ± 17.33 years and a median age of 26 years. Females constituted 57.4% of the study population.

The majority of patients (88.1%) presented directly to the emergency department, while 11.9% were referred from other healthcare facilities for vaccination.

Students represented the largest occupational subgroup (27.8%), followed by individuals categorized as “other” (31.4%), which included multiple small occupational categories. Children accounted for 11.7% of cases. When children and students were combined, younger individuals accounted for 39.5% of the cohort (Table 1).

### 3.2. Exposure-Related Characteristics

Most exposures occurred in public environments. The street was the most common location (44.8%), followed by homes (19.0%) and gardens (15.0%), together accounting for 78.8% of incidents.

Cats were responsible for 86.3% of exposures, while dogs accounted for 13.0%. Exposure via other animal species was rare (Table 2).

Scratching was the predominant mechanism of exposure (81.5%), whereas bites accounted for 18.5%.

The majority of animals were classified as strays (90.1%). The vaccination status of the animal was unknown in 81.2% of cases. Post-exposure animal observation was documented in only 20.2% of incidents.

Pre-exposure rabies prophylaxis was absent in 90.2% of patients. Most individuals (74.5%) presented to the emergency department within 24 h of exposure.

Nearly all injuries were superficial (99.8%), with deep injuries observed in only 0.2% of cases (Table 2).

### 3.3. Post-Exposure Clinical Management

Wound care was performed in 97.8% of patients. Rabies vaccination was initiated in 98.8% of cases.

A four-dose vaccination protocol was recommended for 91.2% of patients. Completion of the full four-dose schedule was documented in 73.7%, whereas 7.3% did not complete the vaccination series.

Rabies immunoglobulin (RIG) was administered in 0.6% of cases.

Tetanus prophylaxis was provided in 71.4% of patients.

No patient required surgical intervention. All patients were discharged from the emergency department. No mortality or major complications were observed during the 30-day follow-up period (Table 3).

Superficial injuries were defined as minor scratches or abrasions without deep tissue involvement (broadly corresponding to WHO Category II exposure), whereas deep injuries referred to transdermal bites or wounds involving deeper tissue layers (comparable to WHO Category III exposure). Open area refers to public outdoor spaces such as parks, squares, or other open areas not classified as streets, homes, or gardens. Observed animals were monitored for 10 days in accordance with national rabies management guidelines.

## 4. Discussion

This study outlines the demographic, clinical, and management characteristics of 1960 patients who visited the emergency department of a large metropolitan hospital with suspected animal exposure over a six-month period.

The predominance of young adults and students suggests that exposure to animals is more common among socially active individuals in urban settings. The findings provide valuable insights into the regional epidemiology of rabies-suspected cases and the clinical reasoning behind post-exposure prophylaxis (PEP) decisions in an urban environment [25]. Similarly high volumes of suspected rabies exposures requiring prophylaxis have been reported in regions where rabies is endemic, with PEP administration greatly surpassing the number of confirmed rabies cases [26,27,28], highlighting the need to assess real-world prophylaxis practices within their structural and epidemiological contexts.

The demographic distribution indicates that animal exposure in metropolitan environments is not confined to children alone. Although children are traditionally considered a high-risk group due to their closer, often uncontrolled contact with animals [26], our data demonstrate that exposure risk spans all age groups. When students and children are considered together, younger individuals account for approximately 40% of cases. This distribution likely reflects the normalization of human–animal interaction in densely populated urban environments rather than isolated vulnerability within a single demographic group.

A defining epidemiological feature of this cohort was the predominance of cat-related exposures (86.3%). While dog-mediated transmission accounts for most human rabies deaths globally, exposure dynamics vary substantially according to ecological and sociocultural conditions [29]. In metropolitan areas of Türkiye, free-roaming cats are deeply integrated into daily urban life, occupying residential complexes, public spaces, and transportation hubs. Such coexistence increases the likelihood of minor defensive injuries, particularly scratches. Comparable exposure profiles have been reported in Mediterranean and Middle Eastern urban settings, where cats are a leading source of suspected rabies exposure despite having lower documented transmission rates than dogs [8,30,31,32]. These findings highlight the importance of contextualizing rabies epidemiology within local ecological realities rather than extrapolating solely from global transmission patterns. Although dogs remain the primary reservoir of rabies in many regions, cats may also act as secondary vectors and contribute to human exposures, particularly in urban environments where human–cat interactions are frequent. The clinical severity profile in this study was overwhelmingly mild. Nearly all injuries were superficial (99.8%), and rabies immunoglobulin was required in only 0.6% of patients.

In this study, the term “biological risk of transmission” was used to describe the potential likelihood of rabies virus transmission based on characteristics of the exposure, including the type of contact, the depth of the injury, and the species of the animal involved. However, rabies vaccination was initiated in 98.8% of patients. This contrast between low wound severity and high vaccination frequency represents a central tension in urban rabies management. Importantly, this pattern does not necessarily indicate inappropriate prophylaxis; rather, it reflects clinical decision-making under conditions of limited certainty. However, it should be noted that even minor scratches may theoretically be associated with rabies transmission if contaminated with infected saliva, and therefore, superficial injuries should not be considered completely risk-free.

In this cohort, 90.1% of animals were classified as strays; vaccination status was unknown in 81.2% of cases; and post-exposure observation was documented in only 20.2% of cases. Established international and national guidelines recommend observing the implicated animal when feasible to guide rational PEP administration and potentially avoid unnecessary vaccination [33,34]. In metropolitan settings characterized by large populations of free-roaming animals and fragmented veterinary follow-up systems, systematic observation is often impractical. Under such structural constraints, clinicians may reasonably adopt a precautionary approach given the uniformly fatal outcome of untreated rabies. Accordingly, high vaccination rates in this context appear to be shaped more by surveillance limitations and infrastructural realities than by wound severity alone.

From a public health perspective, the cumulative impact of frequent suspected exposures is considerable. Previous studies from rabies-endemic regions have reported that the number of individuals receiving PEP far exceeds the number of confirmed rabies cases, generating a significant operational and economic burden [26,27,30]. Although this study did not include a formal cost analysis, the near-universal vaccination rate observed among predominantly superficial injuries suggests that improvements in animal vaccination registries, documentation systems, and coordination between healthcare and veterinary authorities may support more refined exposure assessment and more efficient resource utilization [35].

Timeliness of presentation is another key determinant of effective rabies prevention. In this study, 74.5% of patients presented within 24 h of exposure, indicating adequate awareness and access to emergency services in an urban setting. Early presentation aligns with recommended prophylaxis timing and likely contributed to the absence of adverse outcomes. Nevertheless, delayed presentation in approximately one-quarter of cases remains clinically relevant, particularly given the time-sensitive nature of rabies prophylaxis [36].

Vaccination practices were largely consistent with recommendations from the World Health Organization and the Ministry of Health of the Republic of Türkiye [37]. However, incomplete adherence to scheduled vaccination doses among a subset of patients suggests that follow-up mechanisms may need strengthening. Integration with primary healthcare systems, reminder messaging strategies, and digital tracking tools may enhance adherence and optimize completion rates [38,39].

The absence of severe tissue injury requiring surgical intervention and the lack of mortality during follow-up further confirm that the majority of exposures in this cohort were low-to-moderate risk. These favorable outcomes support the effectiveness of current management practices and reflect the predominantly superficial nature of injuries.

In conclusion, this study demonstrates that rabies management in metropolitan settings in Turkey is shaped by the interplay between ecological characteristics, limited animal surveillance capacity, and precautionary clinical decision-making. In urban regions with high densities of free-roaming animals, maintaining patient safety while promoting rational PEP utilization requires context-sensitive risk stratification supported by strengthened intersectoral collaboration within a One Health framework.

## 5. Limitations

This study has several limitations. First, its retrospective design depends on the accuracy and completeness of electronic medical records. Although standardized hospital documentation systems were used, misclassification or incomplete recording of exposure details cannot be entirely excluded. Second, the study was conducted at a single tertiary metropolitan center, which may limit the generalizability of the findings to rural settings or regions with different animal population dynamics and healthcare access structures.

Third, reliable information regarding animal vaccination status and post-exposure observation was limited, particularly for free-roaming animals. This reflects real-world surveillance constraints and may have affected risk stratification processes. Fourth, clinical outcomes were assessed within the available follow-up period, and long-term adherence to vaccination schedules beyond documented visits could not be fully evaluated.

Finally, although this study provides a comprehensive descriptive analysis of urban animal exposures, it does not include formal cost analysis or comparative evaluation of alternative prophylactic strategies. Future multicenter prospective studies that integrate veterinary and public health surveillance systems may enable a more detailed assessment of the rational use of PEP in metropolitan settings.

## 6. Conclusions

This study shows that urban animal exposures in a large metropolitan setting are predominantly superficial and primarily associated with cats, yet are accompanied by near-universal rabies vaccination. The coexistence of low-severity injuries, limited animal observation, and uncertain vaccination status illustrates the structural challenges of rabies risk assessment in densely populated urban environments.

Although the biological transmission risk appeared low in most cases, precautionary prophylaxis was frequently implemented, reflecting surveillance constraints and the uniformly fatal nature of untreated rabies. These findings highlight that rabies management in metropolitan settings is shaped not only by wound severity but also by infrastructural and monitoring limitations.

Sustainable rabies control in regions with high densities of free-roaming animals requires strengthened surveillance systems, improved documentation and risk stratification, and closer coordination between healthcare and veterinary sectors. Context-sensitive strategies that balance patient safety with rational PEP utilization are essential for effective urban rabies prevention.

## Figures and Tables

**Table 1:** Demographic characteristics of the study population (n = 1960).

	n (%)/Mean ± SD	%/Median (Min.–Max.)
Age	28.79 ± 17.33	26 (0–89)
Sex Female Male	1126 (57.4) 834 (42.6)	57.4 42.6
Mode of presentation Primary presentation Referred from another healthcare facility for vaccination	1727 (88.1) 233 (11.9)	88.1 11.9
Occupation Child Student Worker Officer Retired Housewife Other	229 (11.7) 544 (27.8) 103 (5.3) 285 (14.5) 93 (4.7) 90 (4.6) 616 (31.4)	11.7 27.8 5.3 14.5 4.7 4.6 31.4

Mean: average; SD: standard deviation; min: minimum; max: maximum.

**Table 2:** Characteristics of animal exposures and injury types (n = 1960).

	n (%)
Location of incident Home Garden Street Open area Forest Other	372 (19) 293 (15) 877 (44.8) 7 (0.4) 6 (0.3) 403 (20.6)
Animal involved Cat Dog Rabbit Mouse Horse Monkey Bat Other	1691 (86.3) 254 (13) 2 (0.1) 3 (0.2) 3 (0.2) 4 (0.2) 2 (0.1) 1 (0.1)
Mechanism of exposure Bite Scratch	363 (18.5) 1597 (81.5)
Pre-exposure prophylaxis No Yes	1767 (90.2) 193 (9.8)
Animal ownership No (stray) Yes (owned)	1766 (90.1) 194 (9.9)
Vaccination status of the animal Not vaccinated Vaccinated Unknown	340 (17.3) 29 (1.5) 1591 (81.2)
Outcome of the animal Alive Euthanized Died Unknown	66 (3.4) 4 (0.2) 4 (0.2) 1886 (96.2)
Observation of the animal after exposure: Not observed Observed	1565 (79.8) 395 (20.2)
Time to presentation after exposure <24 h >24 h	1461 (74.5) 499 (25.5)
Type of injury Superficial Deep	1956 (99.8) 4 (0.2)

**Table 3:** Clinical management and post-exposure prophylaxis after animal exposure.

	n %
Wound care Not performed Performed	43 (2.2) 1917 (97.8)
Post-prophylaxis rabies vaccination Not administered Administered	24 (1.2) 1936 (98.8)
Recommended vaccine doses 0 2 4	22 (1.1) 151 (7.7) 1787 (91.2)
Vaccine doses received by the patient 0 1 2 3 4	23 (1.2) 90 (4.6) 237 (12.1) 166 (8.5) 1444 (73.7)
Rabies immunoglobulin Not administered Administered	1949 (99.4) 11 (0.6)
Tetanus prophylaxis No Yes	561 (28.6) 1399 (71.4)
Surgical intervention No	1960 (100)
Outcome Discharged from the emergency department	1960 (100)

## Data Availability

The datasets used and/or analyzed during the current study are available from the corresponding author upon reasonable request.
